# Symptomatic Porencephalic Cyst in the Chronic Postoperative Phase Following Evacuation of Cerebellar Hemorrhage: A Case Report

**DOI:** 10.7759/cureus.92004

**Published:** 2025-09-10

**Authors:** Songhyon Lee, Katsunari Kiko, Takuya Moriwaki

**Affiliations:** 1 Neurological Surgery, Asahi General Hospital, Asahi, JPN

**Keywords:** adult case, cerebellar hemorrhage, chronic phase, porencephalic cyst, post-surgery

## Abstract

Porencephalic cysts are typically observed in fetal or pediatric patients and are rarely reported in adults. While some cases have been reported in the acute or subacute phase following intracerebral hemorrhage, delayed formation during the chronic phase is exceedingly uncommon, particularly in the posterior fossa. We report the case of a 53-year-old woman who developed progressive ataxia and dysarthria eight months after surgical evacuation of a cerebellar hemorrhage. Imaging revealed a 55-mm CSF-filled cyst in the left cerebellum. She underwent cyst wall fenestration and opening of the cisterna magna, which resulted in gradual cyst shrinkage and symptom resolution. No recurrence was observed at the five-month follow-up. Only four cases of porencephalic cyst formation following hematoma evacuation have been reported, all in the supratentorial region and during the acute or subacute phase. This case is the first to describe delayed symptomatic cyst formation in the cerebellum. The clinical course suggests a check-valve mechanism as the underlying pathophysiology. This case highlights the importance of considering delayed porencephalic cyst formation in the differential diagnosis of new neurological symptoms during the chronic postoperative phase. It underscores the value of long-term imaging surveillance after parenchymal brain injury.

## Introduction

Porencephalic cysts are rare, CSF-filled cavities within the brain's parenchyma [1]. They are generally classified as congenital or acquired. Congenital porencephalic cysts are often associated with developmental brain anomalies or insults during the fetal period, such as brain aplasia, and some cases have been linked to genetic mutations in genes like COL4A1 [2,3]. The estimated incidence for congenital forms is approximately 3.5 per 100,000 live births, making it a condition primarily encountered in pediatric populations [4]. Acquired porencephalic cysts, conversely, develop following postnatal brain injury, such as infection, trauma, and ischemic stroke or hemorrhage [1,5]. While cyst formation after surgical hematoma evacuation is a known complication, the existing literature almost exclusively reports these developing in the acute or subacute phase [6-10]. The emergence of a symptomatic, expanding cyst in the chronic phase, months or years after the initial event, is exceptionally rare, leaving a gap in the literature regarding its management.

The pathophysiology of delayed cyst expansion remains debated, with proposed theories including a check-valve mechanism allowing unidirectional CSF influx [8,9] or an osmotic and concentration gradient drawing fluid into the cavity [6,7,9,10]. Here, we present a rare case of a symptomatic porencephalic cyst that developed eight months after the surgical evacuation of a cerebellar hemorrhage. We discuss its clinical implications and management based on a review of the literature.

## Case presentation

**Figure 1:**
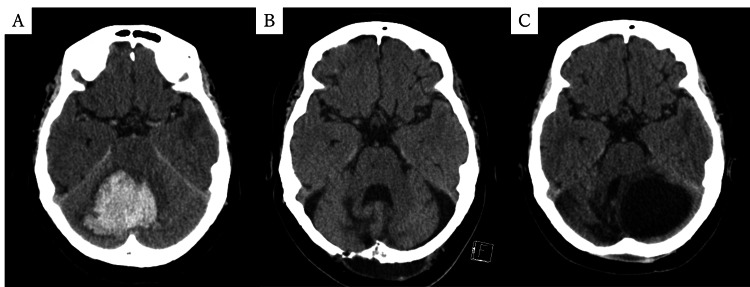
Serial CT images (A) Initial CT showing cerebellar hemorrhage. (B) CT at one month postoperatively, without cyst formation. (C) CT at eight months postoperatively demonstrating a newly developed cystic lesion in the left cerebellar hemisphere.

A 53-year-old woman with no significant past medical history was transported to our hospital with impaired consciousness and was diagnosed with a right cerebellar hemorrhage (Figure 1A). A preoperative CT angiography revealed no evidence of an aneurysm or vascular malformation as a cause for the hemorrhage. She underwent emergency surgical evacuation of the hematoma via a standard midline suboccipital craniotomy. A synthetic dural substitute was used for dural closure. Postoperatively, her consciousness improved, but she had residual cerebellar ataxia. Following a stable postoperative course, her neurological deficits were deemed to require intensive physical therapy, and she was discharged to a rehabilitation facility one month after surgery (Figure 1B).

**Figure 2:**
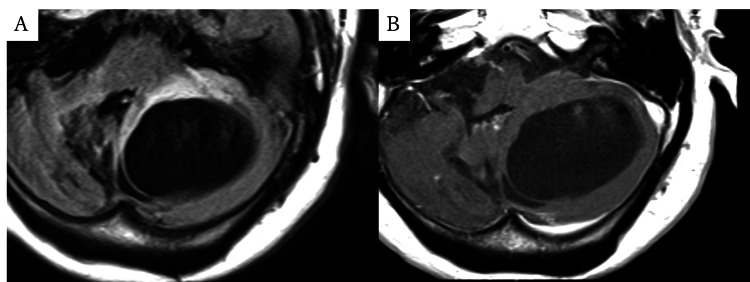
Preoperative MRI (A) FLAIR image showing the cystic lesion isointense to CSF. (B) Contrast-enhanced T1-weighted image showing no enhancement.

Eight months after the initial surgery, she developed progressive gait disturbance and worsening dysarthria. A follow-up CT revealed a 55-mm cystic lesion in the left cerebellum (Figure 1C). She was transferred back to our hospital. On admission, her Glasgow Coma Scale score was E4V5M6. Neurological examination revealed left-sided cerebellar ataxia and dysarthria. MRI demonstrated a cystic lesion isointense with CSF on FLAIR, without contrast enhancement (Figure 2A, 2B). She was diagnosed with a symptomatic porencephalic cyst.

**Figure 3:**
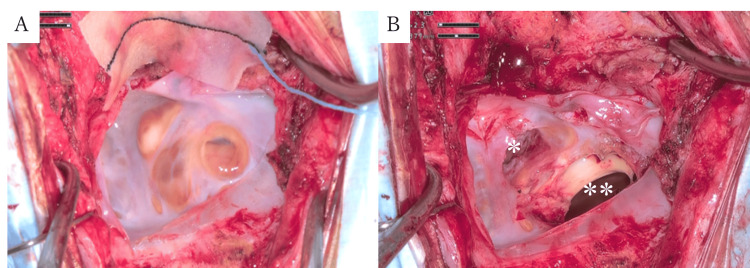
Intraoperative photographs (A) Cyst wall surface exposed. (B) Fenestration of the cyst wall and opening of the cisterna magna ^*^ Cisterna magna ^**^ Cyst cavity

Given the progressive symptoms and significant mass effect, surgical intervention was planned. She underwent a reperation via the previous midline suboccipital approach. Using standard microsurgical techniques, a fenestration of approximately 1 cm in diameter was created in the cyst wall, and the cisterna magna was widely opened to establish communication between the cyst cavity and the cisternal space (Figure 3A, 3B). The cyst contained clear, colorless, CSF-like fluid, with no evidence of hemorrhagic or purulent content. A biopsy specimen taken from the cyst wall demonstrated normal brain tissue.

**Figure 4:**
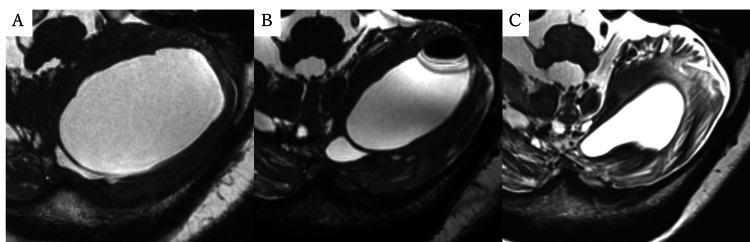
Follow-up heavy T2-weighted MR images demonstrating reduction in cyst size over time (A) At onset of cyst formation (eight months post-op). (B) One month after surgery. (C) Five months after surgery.

The patient’s postoperative course was favorable, with a noticeable improvement in her ataxia and dysarthria. A follow-up MRI five months later demonstrated a marked reduction in the cyst’s size (Figure 4A-4C).

## Discussion

We report a rare case of a delayed, symptomatic porencephalic cyst that developed contralateral to a surgically treated cerebellar hemorrhage. To the best of our knowledge, only five cases of postoperative cyst formation following hematoma evacuation have been previously reported (Table 1) [6-10]. All of these were supratentorial lesions that developed in the subacute phase, between eight and 17 days postoperatively. Our case is distinctly different from these reports in that it was an infratentorial lesion, developed in the chronic phase over eight months, and formed on the contralateral side to the primary hematoma.

**Table 1:** Reported adult cases of porencephalic cyst following evacuation of intracerebral hematoma CPS, cystoperitoneal shunting

Author (year) [reference]	Age/sex	Location	Initial surgery	Secondary surgery	Interval (days)
Miyata et al. (2000) [6]	62/M	Supratentorial	Craniotomy	Drainage	17
Asayama et al. (2015) [7]	82/F	Supratentorial	Craniotomy	Drainage	8
Shinagawa et al. (2020) [8]	69/F	Supratentorial	Craniotomy	Craniotomy	10
Noguchi et al. (2025) [9]	73/F	Supratentorial	Craniotomy	Craniotomy	14
Takamura (2025) [10]	88/M	Supratentorial	Craniotomy	CPS	14
Present case	53/F	Infratentorial	Craniotomy	Craniotomy	Approximately 240

Several mechanisms for postoperative cyst formation have been proposed. The first involves an osmotic and concentration gradient between the hematoma cavity and the ventricle. The post-evacuation cavity contains residual hematoma components, inflammatory cells, and proteins, creating a hyperosmolar state compared to the CSF. This osmotic difference is thought to drive a unidirectional flow of CSF into the cavity, either across the ventricular wall or through a minute communication created during surgery, leading to cyst formation and expansion, as has been suggested in several prior reports [6,7,9,10].

A second proposed mechanism is the presence of a check-valve system that promotes CSF influx while preventing its outflow. In this scenario, CSF pulsations are thought to drive fluid unidirectionally into the cavity through a narrow communication between the ventricle and the cyst, a theory supported by other cases [7-9]. This physical mechanism is considered particularly important in cases where an osmotic gradient alone cannot account for the rapid expansion and mass effect of the cyst [8].

Furthermore, anatomical sequestration of the hematoma cavity is considered a contributing factor. While the cyst may be adjacent to a ventricle, allowing CSF to enter, its communication with other spaces, such as the subarachnoid space, may be obliterated by adhesions. This traps the fluid within the cavity, leading to increased internal pressure and progressive cyst enlargement, as described in one report [10].

In the present case, the slow, progressive enlargement of the cyst over an eight-month chronic course strongly suggests that a check-valve mechanism, rather than an osmotic gradient, was the primary driver of its formation.

Regarding the contralateral lesion, the initial imaging was critical, as it showed the hematoma extending across the midline and causing direct parenchymal injury to the left cerebellar folia. We postulate this direct injury created the nidus for cyst formation. We surmise that a check-valve mechanism subsequently developed, potentially involving arachnoid scarring and the implanted synthetic dural substitute, which acted as a one-way flap and led to the cyst’s progressive expansion. The patient's progressive ataxia and dysarthria were direct consequences of the mass effect exerted by this expanding cyst on the surrounding cerebellar parenchyma. A review of the preoperative MRI confirmed this significant mass effect, although no tonsillar herniation was present.

The standard treatment for a symptomatic porencephalic cyst is surgical decompression. Reported techniques include cyst fenestration [11], cystoperitoneal shunting (CPS) [12], and ventriculocystostomy [13].

In our case, we chose cyst fenestration to the cisterna magna. This decision was based on our hypothesis of a check-valve mechanism. By creating a wide, permanent communication with a major CSF cistern, this technique directly addresses the presumed pathophysiology. In contrast, CPS, while effective, carries the risks of shunt malfunction and infection [14]. Fenestration avoids these device-related complications and offers a more definitive physiological solution when anatomically feasible.

Currently, there is no established gold-standard treatment for this rare condition, making it crucial to tailor the therapeutic strategy to the presumed pathophysiology and anatomical location in each case. While we believe the accumulation of experience from cases like ours is essential, we also acknowledge the limitations of this report. As a single case study, our findings cannot be generalized, and the proposed check-valve mechanism, though clinically plausible, was not definitively proven by the nonspecific histopathological findings. Furthermore, a longer-term follow-up beyond five months is necessary to confirm the lasting efficacy of our chosen intervention. Continued reporting of such cases, with treatment selection guided by pathophysiological insights, is essential to deepen our collective understanding and improve the management of this rare postoperative phenomenon.

## Conclusions

We report a rare case of a symptomatic porencephalic cyst that developed in the chronic phase following the surgical evacuation of a cerebellar hemorrhage. Postoperative cyst formation is typically an acute or subacute event, making the delayed presentation in the chronic period an exceptionally rare finding in the literature.

This case highlights the need for long-term surveillance for cystic changes after the evacuation of large parenchymal hematomas. The emergence of new or progressive neurological deficits, even months after surgery, should prompt immediate neuroimaging to rule out such delayed complications. Timely diagnosis and surgical intervention are crucial for achieving favorable outcomes, as these cysts can exert significant mass effects. This report contributes to the understanding and management of this rare postoperative phenomenon.
